# Hidradenitis suppurativa with disease onset before age 18-years in Brazil: diagnostic delay and adult disease burden^؆^

**DOI:** 10.1016/j.abd.2026.501478

**Published:** 2026-11-09

**Authors:** Priscilla Filippo Alvim de Minas Santos, Luna Azulay-Abulafia, Roberto Souto da Silva, Mario Chaves Loureiro do Carmo, Luciano Alves Favorito

**Affiliations:** a Department of Dermatology, Hospital Universitário Pedro Ernesto, Universidade do Estado do Rio de Janeiro, Rio de Janeiro, RJ, Brazil; b Department of Surgery, Hospital Universitário Pedro Ernesto, Universidade do Estado do Rio de Janeiro, Rio de Janeiro, RJ, Brazil

Dear Editor,

Hidradenitis Suppurativa (HS) is a chronic, recurrent inflammatory disease of the hair follicle characterized by painful nodules, abscesses, tunnels, and scarring in predominantly intertriginous areas, with substantial physical and psychosocial burden.^1^, ^2^ Although HS is usually diagnosed in early adulthood, disease onset frequently occurs during childhood and adolescence, when recognition remains limited and diagnostic delay remains common.^3^, ^4^ Early diagnosis is crucial, as timely intervention may prevent progression to irreversible structural damage and reduce long-term disease burden.^2^, ^4^

Brazilian data on childhood-onset HS remain scarce. Given the potential impact of delayed diagnosis on disease severity and treatment burden, we aimed to characterize the clinical profile and adult outcomes of Brazilian patients with HS whose symptoms started before the age of 18-years.

We conducted a retrospective descriptive study with information based on the electronic medical records of patients followed at a university hospital in Rio de Janeiro, Brazil, between May 2017 and August 2025. The study was approved by the institutional ethics committee (CEP nº6.260.651). Adult patients (≥18-years) with hidradentitis supurativa (HS), whose disease onset occurred before 18-years of age and who had been followed at the specialized outpatient clinic for at least 6-months, were included.

Among 302 patients with HS, 88 (29.1%) reported symptom onset during childhood or adolescence. Of these, 84 who met the inclusion criteria were studied. Information regarding the age of symptom onset was obtained from the reports in the medical records. The mean age at assessment was 26.1-years (SD = 8.35), and most patients were female (71.43%), consistent with the female predominance described in epidemiological studies of HS^3^, ^5^ (Table 1).

**Table 1:** Baseline characteristics of patients with hidradenitis suppurativa included in the study.

Variable	Total
Age, mean ± SD	26.15 ± 8.35
Gender, n (%)	
Male	24 (28.57%)
Female	60 (71.43%)
Skin Color, n (%)	
White	42 (50.00%)
Brown	19 (22.62%)
Black	23 (27.38%)
Current Follow-up, n (%)	
Yes	33 (39.29%)
No	51 (60.71%)
Hurley classification, n (%)	
I	6 (7.14%)
II	30 (35.71%)
III	48 (57.14%)
Hospitalization for HS, n (%)	28 (33.33%)
Comorbidities, n (%)	68 (80.95%)
Smoking, n (%)	7 (8.33%)
Family history of HS, n (%)	12 (14.29%)
Areas affected by HS, n (%)	
Head/neck	1 (1.19%)
Armpit	74 (88.10%)
Chest	22 (26.19%)
Inguinal region	47 (55.95%)
Perineal region	9 (10.71%)
Buttocks	24 (28.57%)
Genital	14 (16.67%)
Systemic treatments used for HS, n (%)	
Sulfamethoxazole with trimethoprim	58 (69.05%)
Metformin	48 (57.14%)
Doxycycline	42 (50.00%)
Oral contraceptive	36 (60.00%)
Cefalexin	29 (34.52%)
Adalimumabe	22 (26.19%)
Clindamycin	19 (22.62%)
Oral corticosteroid	19 (22.62%)
Tetracycline	11 (13.10%)

The mean age at symptom onset was 14-years (range 5–17), whereas the mean age at diagnosis was 17.9-years, resulting in an average diagnostic delay of 43.3-months. Furthermore, 76.2% of patients reported consulting four or more physicians before diagnosis. These findings reinforce that delayed diagnosis remains a major challenge in HS worldwide, even in specialized settings.^4^

Disease severity according to the Hurley classification, refers when patients were evaluated as adults at the moment of data collection), demonstrated a predominance of advanced disease, with 48 patients (57.14%) classified as Hurley stage III, 30 (35.71%) as Hurley stage II, and only 6 (7.14%) as Hurley stage I. This predominance of moderate-to-severe disease may be partially explained by the referral nature of our tertiary specialized HS clinic, which mainly receives complex cases. Additionally, the substantial diagnostic delay observed in our cohort may have contributed to disease progression and the development of irreversible structural changes. These findings are consistent with the high frequency of fistulas, fibrotic bands, and surgical interventions observed in this population.

Obesity was the most frequent comorbidity (58.3%), followed by acne (29.8%), pilonidal disease (14.3%), and polycystic ovary syndrome (10.7% calculated using only female patients). These findings are consistent with previous studies showing a high burden of metabolic and follicular comorbidities in HS.^6^ Family history was reported by only 14.3% of patients, lower than previously reported rates, which may reflect underreporting or population-specific characteristics.^7^

Most patients (67.9%) had involvement of two or more anatomical sites, predominantly axillary and inguinal regions, in agreement with the classic topography of HS.^2^ Pain was reported by all patients, while fistulas (84.5%), erythema (83.3%), fibrotic bands (71.4%), purulent discharge (70.2%), and macrocomedones (61.9%) were frequent findings, indicating substantial inflammatory and structural disease burden.

Of the 84 patients evaluated, 36 (42.85%) reported having received topical and/or oral treatment during childhood/adolescence after being diagnosed with HS. In adulthood, during follow-up, topical treatment was prescribed for all patients, mainly resorcinol (95.2%) and clindamycin (84.5%). Systemic treatment was necessary in 89.3% of cases, reflecting the severity profile of this cohort. Adalimumab was indicated for 40 (47.6%), but only 22 (26.2%) started treatment, mainly due to limited access in the Brazilian public health system. The other patients were unable to access medication. No other immunobiological agent was indicated. In Brazil, adalimumab is approved and has been available in the public health system since 2018, whereas secukinumab and bimekizumab are approved in the country but not available in the public health system. Therapeutic decisions were based on the Brazilian Society of Dermatology Consensus on HS and current European recommendations for HS management.^8^, ^9^

Surgical treatment was necessary in 40.5% of patients, predominantly for axillary disease, with postoperative complications in 7.1%, rates lower than those reported in recent systematic reviews.^10^

There was a significant association between the severity of hidradenitis suppurativa, according to the Hurley classification, and a history of hospitalization for HS (p = 0.0119), inguinal involvement (p = 0.0121), presence of a pilonidal cyst (p = 0.0416), surgery (p = 0.0011), and use of adalimumab (p = 0.0229). Severe disease (Hurley III) was more frequent among hospitalized patients (78.6% vs. 46.4% in non-hospitalized patients), those with inguinal involvement (63.8% vs. 48.6% without inguinal involvement), and those with pilonidal cysts (91.7% vs. 51.4% without pilonidal cysts). Similarly, Hurley III disease was more frequent among patients who underwent surgery (79.4% vs. 42.0%) or received adalimumab (81.8% vs. 48.4%) than among those who did not undergo surgery or receive adalimumab, respectively (Table 2).

**Table 2:** Associations of Hurley Classification and sex with time to diagnosis and follow-up.

Characteristics	Hurley Classification			Value-p^b^	Sex		Value-p^c^
	Mild (n = 6)^a^	Moderate (n = 30)^a^	Severe (n = 48)^a^		Female (n = 60)^a^	Male (n = 24)^a^	
Disease duration, months	112.000 ± 32.373	90.733 ± 95.891	120.729 ± 91.339	0.3667	110.917 ± 96.559	105.583 ± 75.206	0.8090
Time from symptom onset to diagnosis, months	37.333 ± 15.971	30.600 ± 39.102	52.063 ± 73.386	0.3111	39.767 ± 59.928	52.292 ± 63.552	0.3975
Age at symptoms onset, years	14.833 ± 2.041	13.933 ± 2.333	13.938 ± 2.709	0.7077	13.700 ± 2.540	14.750 ± 2.364	0.0848
Age at diagnosis, years	18.167 ± 2.639	16.633 ± 3.222	18.646 ± 5.825	0.2125	17.233 ± 4.890	19.542 ± 4.681	0.0513
Follow-up, months	17.500 ± 27.061	17.633 ± 19.854	21.771 ± 23.975	0.7111	17.700 ± 19.145	25.708 ± 29.297	0.2251

a Mean ± SD.

b ANOVA.

c Student´s *t*-test.

Of the total sample, 51 (60.71%) patients had not attended follow-up consultations within the preceding six months, a finding that may be attributable to several factors: failure to attend scheduled appointments combined with subsequent difficulty in rescheduling, perceived clinical improvement leading patients to reduce the frequency of follow-up visits, or concurrent follow-up at alternative healthcare services.

Our findings demonstrate that nearly one-third of patients in this Brazilian tertiary cohort reported disease onset during childhood or adolescence, reinforcing that early-onset HS is frequent and may remain underrecognized for years. This diagnostic gap contributes to disease progression, increased surgical burden, and worse long-term outcomes.

These findings are particularly relevant in Brazil, where barriers to specialist access and advanced therapies may further amplify disease burden. Improving awareness of HS among pediatricians, dermatologists, and primary care physicians may enable earlier diagnosis and intervention during the therapeutic window of opportunity, potentially preventing irreversible damage, chronic pain, and psychosocial impairment.^2^, ^8^, ^9^

## What is already known about this topic?

Hidradenitis suppurativa is a chronic, recurrent inflammatory disease associated with significant morbidity, diagnostic delay, and a high burden of comorbidities. Early-onset disease has been linked to increased severity and worse outcomes. However, epidemiological and clinical data in adult populations from low- and middle-income countries, including Brazil, remain scarce.

## What does this study add?

This study provides real-world data from a Brazilian cohort, showing that early-onset hidradenitis suppurativa occurs in nearly one-third of adults and is associated with greater disease severity, high comorbidity burden, and frequent need for hospitalization and advanced therapies. These findings highlight the importance of early recognition and multidisciplinary management to reduce long-term morbidity.

## ORCID ID

Luna Azulay-Abulafia: 0000-0002-4698-2009

Roberto Souto da Silva: 0000-0001-7136-6008

Mario Chaves Loureiro do Carmo: 0000-0003-2943-0322

Luciano Alves Favorito: 0000-0003-1562-6068

## Ethics approval statement

The study was approved according to the ethical standards of the hospital’s institutional committee on experimentation with human beings – IRB number: 6.260.651. It has also been registered in the Brazil Platform, Ministry of Health, National Health Council, and National Research Ethics Commission for studies with human beings.

## Financial support

None declared.

## Authors’ contributions

Priscilla Filippo Alvim de Minas Santos: Conceptualization; methodology; investigation; data curation; formal analysis; writing-original draft.

Luna Azulay-Abulafia: Methodology; Formal analysis; writing-review & editing.

Roberto Souto da Silva: Investigation; data collection and review

Mario Chaves Loureiro do Carmo: Investigation; data collection and review.

Luciano Alves Favorito: Supervision; project administration; writing-review & editing.

All authors have read and agreed to the published version of the manuscript.

## Research data availability

The entire dataset supporting the results of this study was published in this article.

## Conflicts of interest

None declared.
